# Longitudinal study of mammary microbiota dynamics and mastitis prevention in Holstein cows by dry-off strategy

**DOI:** 10.3389/fvets.2026.1751837

**Published:** 2026-02-05

**Authors:** Xinyu Wang, Jing Liu, Lin Jiang, Tingting Sun, Xiaolei He, Lijiao Yang, Mingchun Liu, Fei Xu

**Affiliations:** 1 College of Animal Science and Medicine, Shenyang Agricultural University, Shenyang, China; 2 National Feed Drug Reference Laboratories, Institute of Feed Research, Chinese Academy of Agricultural Sciences, Beijing, China

**Keywords:** cephapirin benzathine, dry cow therapy, Holstein cows, longitudinal study, mammary microbiota

## Abstract

**Introduction:**

Conducting a dry-off period during the late lactation phase in dairy cows can reduce the incidence of clinical mastitis both during the dry period and after subsequent calving. The primary dry-off methods include the application of antibiotics alone or in combination with internal teat sealants. A thorough understanding of the mammary gland microbiota composition before and after dry-off is essential for developing scientifically sound dry-off protocols in practical dairy production.

**Methods:**

Five Holstein cows approaching dry-off were selected for this study. The day of calving is designated as Day 0. Milk samples were collected at three time points around the drying period for 16S rRNA gene sequencing to investigate the differences in mammary microbial composition during these stages. Including Group A (−95 to −67 days), Group B (1 to 2 days), and Group C (14 days).

**Results:**

The results showed that compared to Group A, the abundance of *Firmicutes*, *Actinobacteria*, and *Bacteroides*, as well as the genera *Psychrobacter*, *Romboutsia*, *Clostridium sensu stricto 1*, *Turicibacter*, *Corynebacterium*, *Staphylococcus*, *Streptococcus*, and *Pseudonocardia* (all Gram-positive bacteria) in the milk samples of Group B was significantly lower. In addition, the microbial diversity and richness in the milk samples of Groups A and C exhibited highly significant differences compared to those of Group B (*p* ≤ 0.001). However, no significant differences were found in the microbial communities of the milk samples between Groups A and C (*p* > 0.05). Furthermore, the abundance of beneficial bacterial genera such as Lactobacillus was also increased by antibiotic treatment.

**Conclusion:**

This exploratory study preliminarily indicates that a single dose of cefapirin benzathine administered via intramammary infusion before drying-off can effectively reduce the abundance of specific mammary pathogens, including *Staphylococcus*, *Streptococcus*, and *Corynebacterium* (all Gram-positive bacteria). Furthermore, the diversity and richness of the mammary microbiota generally recovered approximately 14 days after calving. These findings provide a temporal framework for the reconstruction of the mammary microbiota in dairy cows following the dry period.

## 1 Introduction

Mastitis is one of the most common diseases in dairy farming, leading to physical, chemical, and bacteriological changes in milk, thereby posing a direct threat to both milk production and cow health. The occurrence of bovine mastitis is influenced by various factors, such as improper milking practices, environmental contamination, and other factors that can lead to infections by pathogens like *Staphylococcus aureus*, *Streptococcus*, and *Escherichia coli*, which may result in either clinical or subclinical mastitis, with subclinical mastitis causing significant potential economic losses (1). Research indicates that during the dry period, changes in mammary epithelial cells reduce the resistance of mammary tissue to external pathogens, making cows more susceptible to mastitis than during the lactation period. In fact, most cases of postpartum mastitis can be traced back to infections acquired during the dry period (2).

Antibiotic resistance is one of the most serious global public health threats (3). The development of resistance is a natural phenomenon, but the use of antibiotics accelerates the spread and evolution of this resistance (4). Currently, various methods exist for drying off dairy cows, including reducing milking frequency, employing antibiotic dry cow therapy (DCT), and utilizing teat sealants (5). Among these, DCT is the most commonly practiced method worldwide. Driven by concerns over antibiotic resistance and breeding costs, some European countries have already taken measures to reduce the use of antibiotics during the dry-off period of dairy cows (54). These measures include selective dry cow therapy, optimized management measures, and the use of alternatives.^1^ However, antibiotics remain the first choice for most farms during the dry-off period of dairy cows. The combination of teat sealants with antibiotics during the dry period significantly decreases the incidence of mastitis in cows both during the dry period and after calving (6). Although the dry period offers advantages in reducing antibiotic use, the issue of bacterial resistance cannot be overlooked. For instance, *Staphylococcus aureus* exhibits high resistance to penicillin, clindamycin, erythromycin, and gentamicin. Its resistance to Ceftiofur is relatively low, but studies have shown a concerning trend of increasing resistance across nearly all antibiotic classes, which undoubtedly complicates the future management of mastitis in cows (7, 8). The emergence and development of bacterial resistance are linked to the farming environment and management practices on dairy farms. Therefore, studying changes in mammary gland microbiota before and after drying off is of great significance for preventing and treating intramammary infections (IMI) in cows during the dry period.

In this exploratory preliminary study, we aimed to investigate the impact of antibiotic use during the dry period on the mammary microbiota of dairy cows and to explore the time required for its recovery. Prior to the experiment, we proposed the hypothesis that antibiotic use during the dry period would significantly reduce both the diversity and richness of the mammary microbiota, and that the microbiota would recover to pre-dry period levels approximately 2 weeks after calving. Accordingly, this study was designed with three sampling time points surrounding the dry period, at which milk samples were collected for subsequent sequencing to obtain and analyze the distribution characteristics of the mammary microbiota at different time points. The goal was to explore the recovery time of the mammary microbiota following antibiotic use. These findings are expected to contribute to the formulation of more effective management strategies for the dry period in practical dairy farming.

## 2 Materials and methods

### 2.1 Grouping of experimental animals, collection of milk samples, and SCC detection

All experimental protocols have been approved by the Ethics Review Committee of the Feed Research Institute of the Chinese Academy of Agricultural Sciences. Standard recommendations from the National Mastitis Council’s Laboratory Handbook on Bovine Mastitis (9) were adhered to for sample collection.^2^ All experiments were conducted in accordance with the ARRIVE guidelines. The experimental protocol specifies the inclusion of female Holstein cattle, aged between 36 and 48 months and weighing 600–700 kg, who are approaching the dry period. All experimental cows in this study were randomly selected from a healthy herd. All cows enrolled in the trial were clinically healthy with no signs of mastitis—including abnormal milk secretion, udder swelling, fever, or pain upon palpation. The average somatic cell count (SCC) of the included cows was less than 150,000 cells/mL, and no common clinical mastitis pathogens such as *Staphylococcus aureus* or *Streptococcus* spp. were isolated from milk samples cultured on appropriate selective media. To ensure the uniformity and reproducibility of the study, the left front quarter of each cow was selected as the representative for the study. Participants in this study are selected based on these criteria. Throughout the trial, any cows that deviate from the established protocol will be excluded. This includes animals that are either culled or administered alternative antibiotics. Consequently, the data from these excluded cows will not be included in the analysis and discussion of this study. The experimental animals selected in this study can to some extent represent the general situation of dairy cattle breeding in the region. For example, their breeding environment is the most common confined feeding model in the locality, the breed is one of the main breeds in the region, and the principle of random selection of experimental animals was followed during the trial period. Subsequent research can increase the number of experimental animals to validate the research results.

**Figure 1:**
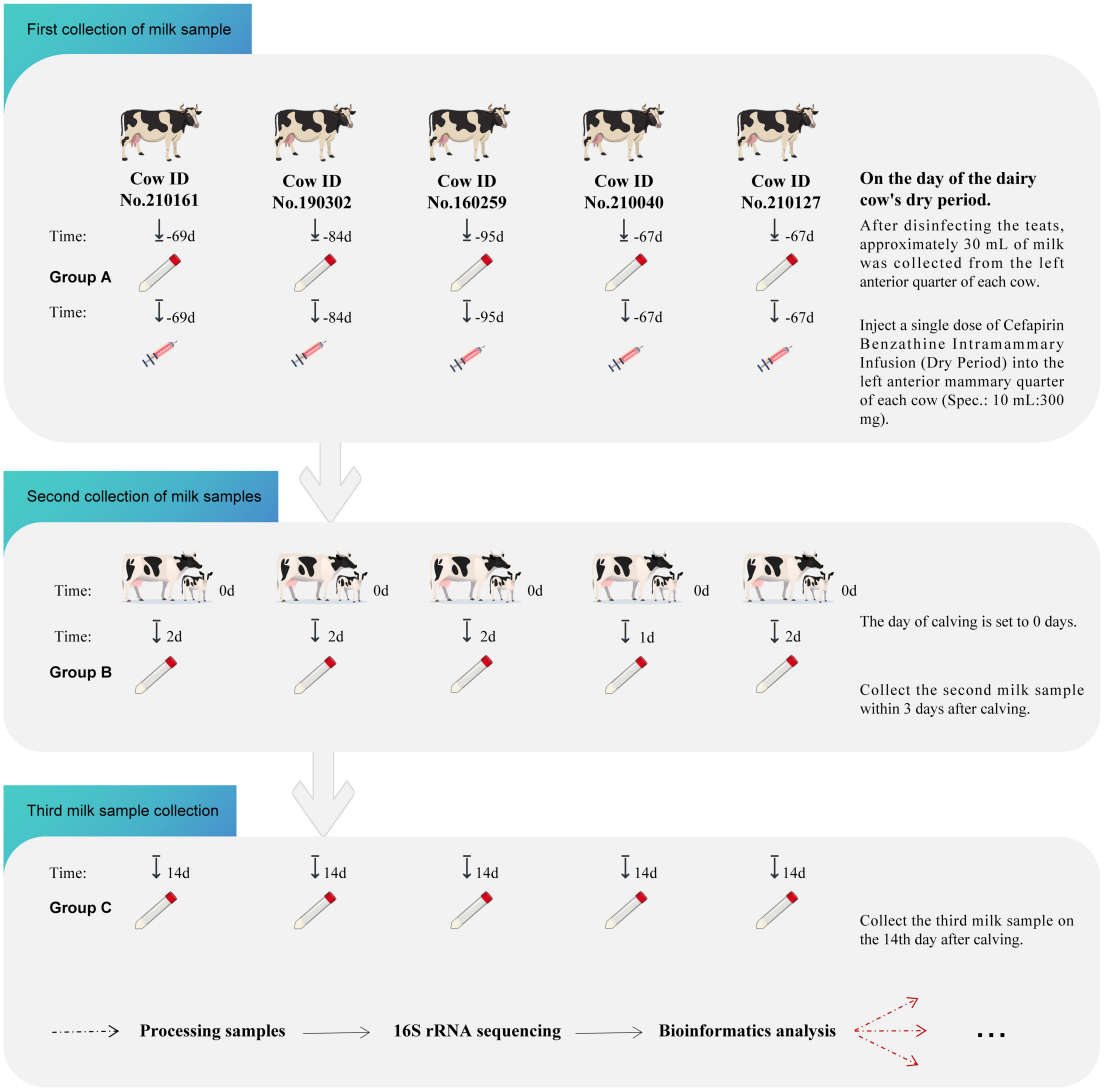
Sample collection workflow. The figure includes information such as cow identification numbers, administration times and doses, sampling times for the three collections, and grouping information. The time of calving is set as Day 0, with “-69” indicating sampling and administration conducted 69 days before calving. “14” refers to the sampling conducted on the 14th day post-calving.

Five multiparous Holstein cows (parity > 2) nearing the dry period were selected from a commercial dairy farm in Tongzhou District, Beijing, China. Milk samples were collected from the left front quarter of each cow, with a total of three samples taken from each cow before and after drying off. The dosing regimen consisted of a single dose of cephapirin benzathine, administered via intramammary infusion prior to drying off. Figure illustrates the specific workflow for sample collection (Figure 1). Before milking, rinse the nipples and dry them with a sterile towel. To avoid contamination from non-mammary bacteria in the teat canal, the first few streams of milk were discarded and then approximately 30 mL of milk from the left front quarter of each cow was collected. The samples were then placed in insulated containers and quickly transported to the GCP Laboratory at the Institute of Feed Research, Chinese Academy of Agricultural Sciences, where Somatic Cell Count (SCC) was measured using a somatic cell counter (Delaval). Subsequently, the milk samples were stored at −80 °C for subsequent analysis.

A total of 15 samples were collected, grouped, and numbered according to sampling times. The first sampling was performed on the day the dry cow treatment was administered, with samples collected before medication administration and designated as Group A. The second sampling was conducted within 3 days post-calving, with samples designated as Group B. The third sampling was conducted on the 14th day post-calving, with these samples designated as Group C.

### 2.2 PCR amplification of the V3–V4 regions of the 16S rRNA gene was performed

Genomic DNA was extracted from milk samples using a commercial DNA extraction kit, and its quality was assessed by 1% agarose gel electrophoresis. To analyze bacterial diversity, the V3–V4 hypervariable regions of the 16S rRNA gene were amplified by polymerase chain reaction (PCR) on an ABI GeneAmp® 9700 thermal cycler using the forward primer 338F (5′-ACTCCTACGGGAGGCAGCA-3′) and the reverse primer 806R (5′-GGACTACHVGGGTWTCTAAT-3′). Negative controls were included in all PCR runs to monitor potential contamination. Each sample was amplified in triplicate; the resulting PCR products were pooled and examined on a 2% agarose gel before being extracted using the AxyPrep DNA Gel Recovery Kit. The purified products were quantified with the QuantiFluor™-ST Blue Fluorescence Quantification System (Promega Corporation). Sequencing libraries were constructed from the amplicons using the NEXTFLEX Rapid DNA Seq Kit, which included adapter ligation and removal of adapter-dimers via magnetic bead–based purification. High-throughput paired-end sequencing was performed on an Illumina NextSeq 2000 platform, targeting a fragment length of approximately 460 bp, to obtain the final sequence data.

### 2.3 Bioinformatics analysis

The raw sequencing data obtained were subjected to quality control using faster and assembled using FLASH to obtain optimized sequences. Noise reduction was performed using DADA2 to correct erroneous sequences and remove chimeric sequences. Valid sequences were clustered at 100% similarity to obtain ASVs (Amplicon Sequence Variants) representative sequences. The classify-sklearn method (Naive Bayes) was employed for taxonomic analysis of the ASVs representative sequences, utilizing the Silva database for alignment, with a classification confidence threshold of 0.7 to derive microbial composition, abundance, and annotation information at the domain, kingdom, phylum, class, order, family, genus, and species levels for each sample. Alpha diversity analysis was conducted using mothur (version v.1.30.1) on the microbial communities in the milk samples from Groups A, B, and C, using the Chao index to reflect species richness and the Shannon index to reflect species diversity. One-way repeated measures analysis of variance was used to assess differences in microbial abundance and diversity. Beta diversity analysis, which reflects the similarity of microbial composition between samples, was performed using the vegan package (version 2.6-4) in R and evaluated by the Bray-Curtis distance algorithm combined with Principal Coordinates Analysis (PCoA). A Circos plot was used to display microbial composition and abundance information in the milk samples of different groups, with bacterial genera accounting for less than 1% of the abundance grouped as others. The ANCOM-BC2 method was applied to analyze abundance differences across each pair of groups, with false discovery rate (FDR) correction. A linear mixed-effects model was fitted, specifying sampling time point as the fixed effect and cow ID as the random effect, to account for non-independence among repeated samples from the same animal. Differences in each bacterial genus between every two groups were further evaluated using the Wilcoxon rank-sum test.

## 3 Results

### 3.1 Sequencing data of milk samples and somatic cell count (SCC) analysis

After sequencing and optimizing 15 samples, a total of 1,255,986 high-quality sequences were obtained, with an average length of 421 bp. Clustering these sequences at 100% similarity yielded 34 phyla, 81 classes, 192 orders, 376 families, 865 genera, 1,391 species, and 6,313 Amplicon Sequence Variants (ASVs). The table displays the somatic cell count (SCC) data (Table 1). In this study, milk samples with somatic cell counts below 200,000 cells/mL were considered healthy, and the findings indicate that all cows remained in a healthy state across all three sampling points. In addition, our research group’s early established ultra-high performance liquid chromatography tandem mass spectrometry (UPLC-MS/MS) detection method was used to detect milk samples, and the results showed that there were no antibiotic residues in the milk samples in this study.

**Table 1:** Somatic cell count (SCC) results of milk samples collected from cows at various time points.

Cow ID	Sample collection		
	−67d to −95d^a^	1d to 2d	14d
ID No. 210161	5.3 × 10^4^^b^	8.3 × 10^4^	7.2 × 10^4^
ID No. 190302	17 × 10^4^	4.6 × 10^4^	3.3 × 10^4^
ID No. 160259	35 × 10^4^	3.7 × 10^4^	9.2 × 10^4^
ID No. 210040	6.1 × 10^4^	7.4 × 10^4^	5 × 10^4^
ID No. 210127	4 × 10^4^	8.1 × 10^4^	6.8 × 10^4^

a The day of calving is defined as day 0. The period from 67 to 95 days prior to calving is denoted as −67d to −95d. All other details remain consistent.

b Somatic cell count results are expressed as cells per milliliter (cells/mL).

### 3.2 Analysis of microbial community diversity

**Figure 2:**
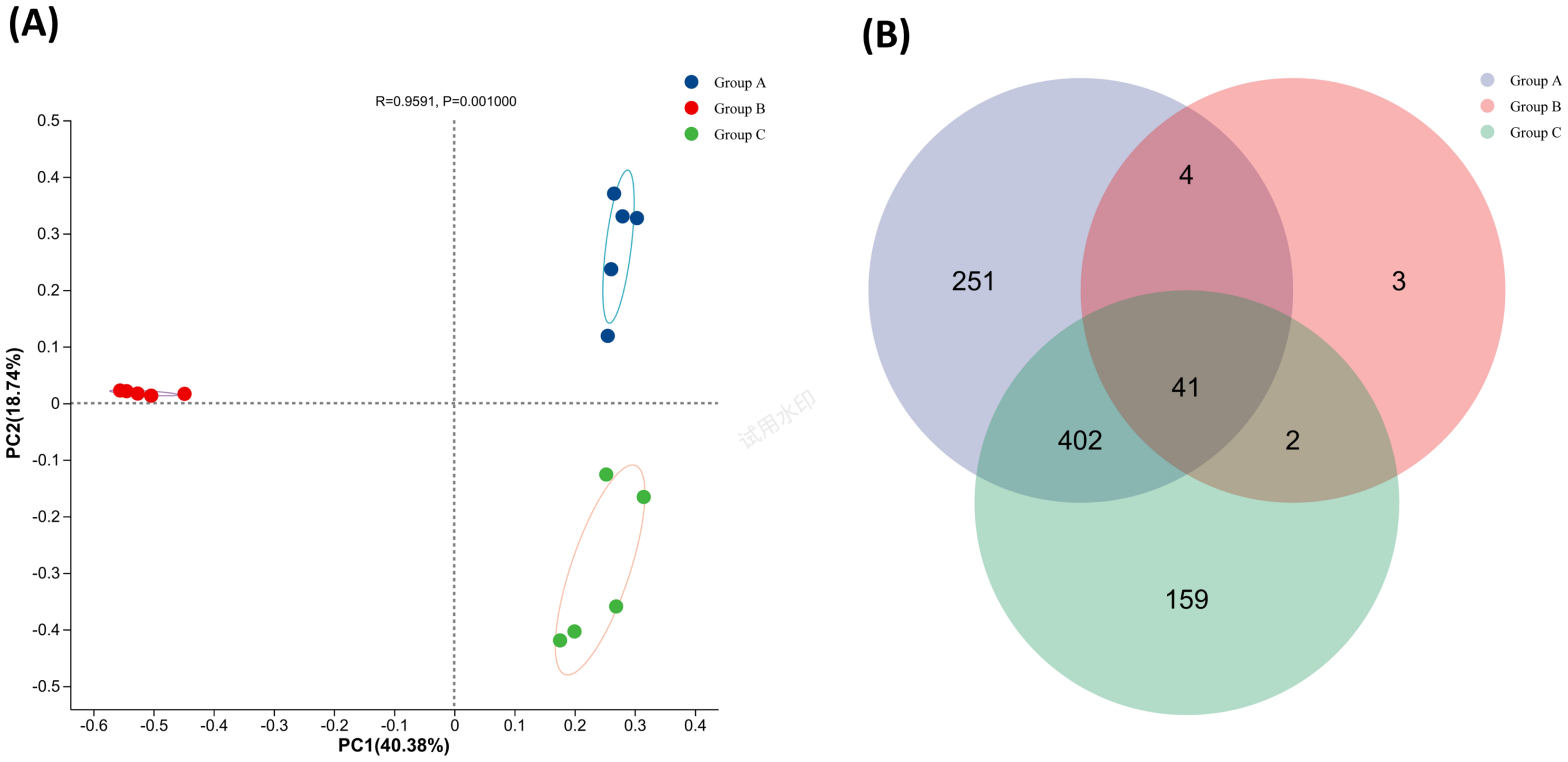
**(A)** β-Diversity analysis. **(B)** Bacterial genus-level Venn diagram.

Compared to Group B, both the Chao and Shannon indices of microbial communities in the milk samples from Groups A and C exhibited extremely significant differences (*p* ≤ 0.001), while no significant differences were observed between the microbial communities of milk samples from Groups A and C (Table 2). This suggests that the richness and diversity of microbial communities in milk significantly decreased following the administration of dry-cow therapy, but showed a marked recovery approximately 14 days after calving, returning to levels comparable to those before treatment. In the Principal Coordinates Analysis (PCoA), the points within each group clustered closely together, indicating a high degree of similarity among samples within the same group. Principal Coordinate 1 (PC1) accounted for 40.38% of the variation. The distance on the PC1 axis between the microbial communities of milk samples from Groups A and C, compared to Group B, showed extremely significant differences (*p* ≤ 0.001), suggesting structural variations between the microbial communities of milk samples from Groups A and C relative to those from Group B (Figure 2A). From the perspective of bacterial genus enrichment, Groups A and C exhibited relatively similar quantities, while Group B showed reduced enrichment of bacterial genera (Figure 2B).

**Table 2:** Alpha diversity of microbial communities in milk samples.

		Group		
		Group A	Group B	Group C
Richness indices	Ace	380.43 ± 101.34***	22.29 ± 10.89	299.73 ± 98.71^▲▲▲^
	Chao	378.77 ± 100.36***	21.75 ± 10.28	300.87 ± 100.72^▲▲▲^
Diversity indices	Shannon	3.9 ± 0.22***	1.07 ± 0.35	4.13 ± 0.47^▲▲▲^
	Simpson	0.05 ± 0.01***	0.46 ± 0.18	0.05 ± 0.03^▲▲▲^

Between A and B in comparison: *0.01 < *p* ≤ 0.05, **0.001 < *p* ≤ 0.01, ***p ≤ 0.001; Between C and B in comparison: ^▲^0.01 < *p* ≤ 0.05, ^▲▲^0.001 < *p* ≤ 0.01, ^▲▲▲^*p* ≤ 0.001; Between A and C in comparison: ns (not significant) indicate (*p* > 0.05).

### 3.3 Analysis of microbial community composition

**Figure 3:**
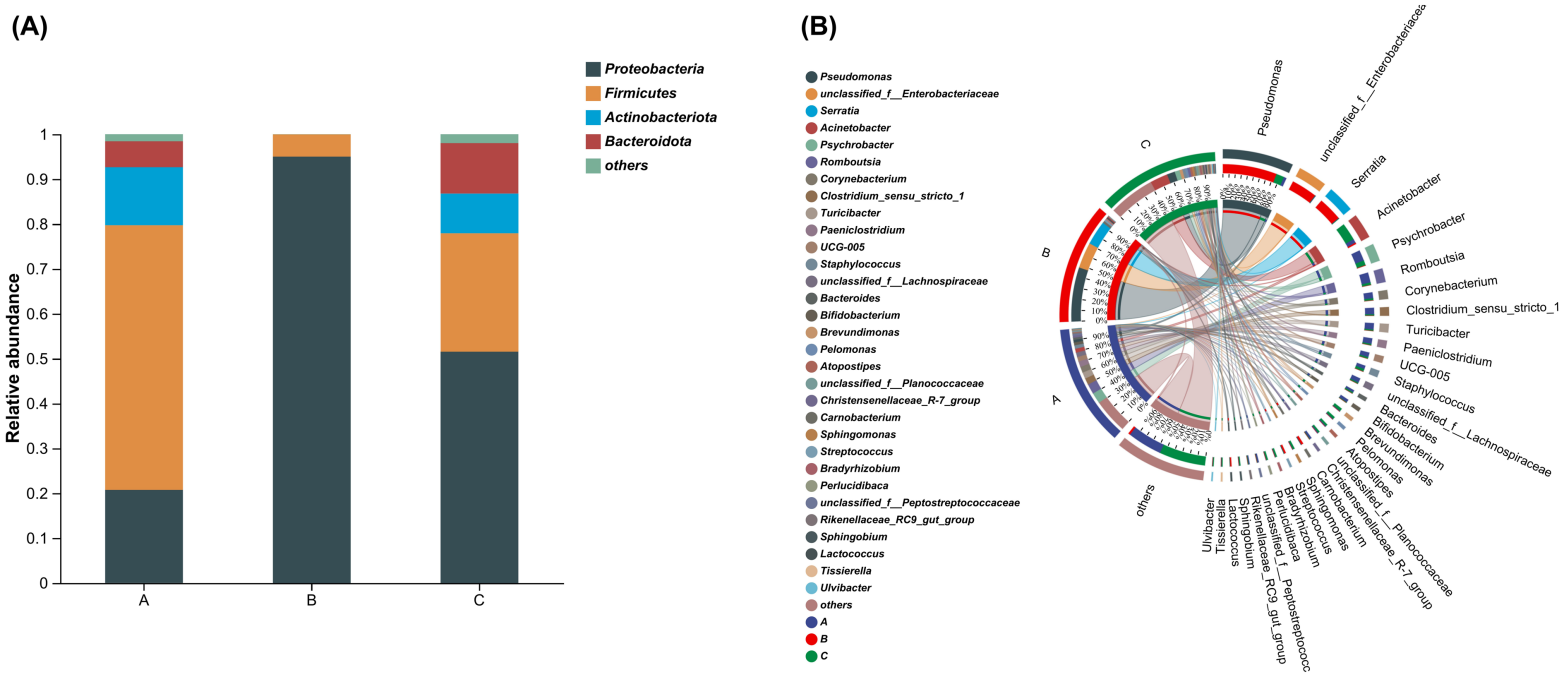
Analysis of microbial composition at the phylum and genus levels. **(A)** The community bar plot illustrates the abundance proportions at the phylum level, with the horizontal axis representing group names and the vertical axis indicating the proportion of each species within the samples of that group. In this study, each group consists of 5 samples, and the plot displays their mean values. **(B)** The Circos plot presents abundance proportions at the genus level; the left semicircle indicates the species composition within each group of samples, while the right semicircle shows the distribution proportions of species across different group samples at this taxonomic level.

At the phylum level, the community composition was analyzed (Figure 3A). Bacterial phyla with abundances of less than 1% were categorized as “Others.” A total of four bacterial phyla were identified: *Proteobacteria*, *Firmicutes*, *Actinobacteria*, and *Bacteroidota*. In the samples from Group A, *Firmicutes* exhibited the highest abundance (60%), followed by *Proteobacteria* (21%), *Actinobacteria* (13%), and *Bacteroidota* (6%). In the samples from Group B, *Proteobacteria* was the predominant phylum (95%), followed by *Firmicutes* (5%). In the samples from Group C, the abundance proportions of the phyla were as follows: *Proteobacteria* (52%), *Firmicutes* (26%), *Bacteroidota* (11%), and *Actinobacteria* (8%).

At the genus level, the community composition was analyzed (Figure 3B). In Group A milk samples, there were 16 bacterial genera with abundances greater than 1%. The genera with the highest abundances included *Psychrobacter* (9.6%), *Romboutsia* (8.9%), *Clostridium sensu stricto 1* (5.7%), *Turicibacter* (5.6%), and *Corynebacterium* (5.3%), among others. In Group B milk samples, 7 bacterial genera exceeded 1% abundance, with *Pseudomonas* (48%) as the most abundant genus, followed by unclassified *Enterobacteriaceae* (22.9%) and *Serratia* (21.5%). Other genera with abundances greater than 1% included *Carnobacterium* (2%), *Streptococcus* (1.9%), *Lactococcus* (1.3%), and *Acinetobacter* (1%). In Group C milk samples, 22 bacterial genera exhibited abundances greater than 1%, with *Acinetobacter* (15.7%) as the most abundant, followed by *Pseudomonas* (7%), *Psychrobacter* (4.3%), *Brevundimonas* (2.6%), and *Staphylococcus* (2.3%), among others.

### 3.4 Analysis of differences in microbial species

**Figure 4:**
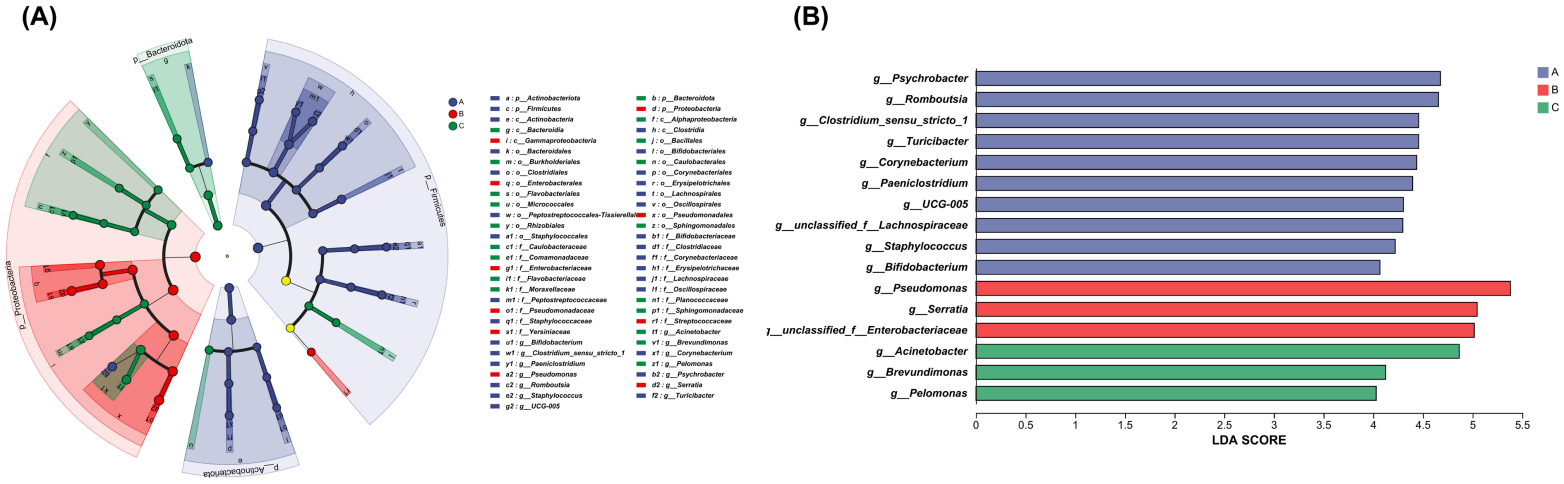
**(A)** The LEfSe analysis of microbial markers across three groups of milk samples is presented. Nodes of different colors in the cladogram represent microbial taxa that are significantly enriched in the corresponding groups and contribute significantly to intergroup differences. Light yellow nodes indicate microbial taxa that show no significant difference across groups or have no significant effect on intergroup differences. **(B)** The LDA bar chart statistically identifies microbial taxa with significant roles among multiple groups, with LDA scores derived from LDA analysis (linear discriminant analysis). Higher LDA scores suggest a greater influence of species abundance on differential effects. The figure displays taxa with LDA scores greater than 4.

The LEfSe analysis results indicate that there are 16 species with significantly different relative abundances (LDA Score > 4, *p* < 0.05) among the three groups of samples (Figures 4A,B). Among these, 10 differentially abundant species were significantly enriched in the milk samples of Group A, primarily represented by genera such as *Psychrobacter*, *Romboutsia*, *Clostridium sensu stricto 1, Turicibacter*, and *Corynebacterium*. Group B exhibited 3 differentially abundant species, which included *Pseudomonas, Serratia*, and *unclassified Enterobacteriaceae*. In Group C, there were also 3 differentially abundant species, with *Acinetobacter* being the most significant and *Brevundimonas* following closely behind. It should be noted that the present results only demonstrate differences in species abundance; the specific biological functions of these differential species and their associations with dry cow therapy and post-calving recovery remain to be further validated through functional omics or *in vitro* experiments.

**Figure 5:**
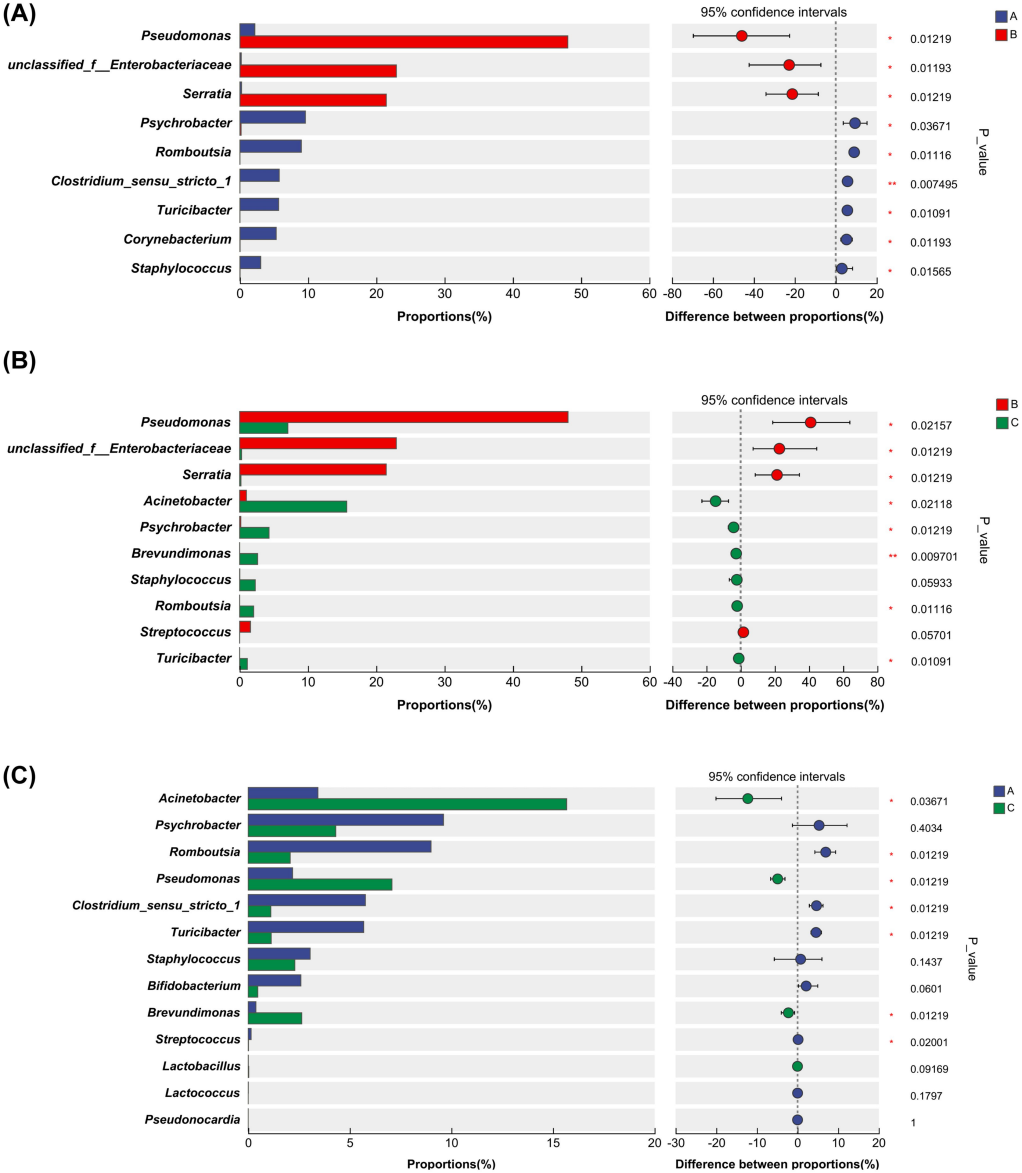
**(A)** Comparison of bacterial genus levels between Group A and Group B. **(B)** Comparison of bacterial genus levels between Group B and Group C. **(C)** Comparison of bacterial genus levels between Group A and Group C. Differences in microbial genus levels among the groups were analyzed by comparing the abundance of bacterial genera between Groups A, B, and C. The bacterial genera listed are based on the results of the LEfSe analysis, as well as additional genera that warrant discussion. The asterisks on the right indicate significant differences, with *p*-values from a Wilcoxon rank-sum test as follows: *0.01 < *p* ≤ 0.05, **0.001 < *p* ≤ 0.01, ***p ≤ 0.001.

Additionally, a differential analysis of the microbial communities between any two groups among the three groups (A, B, and C) of milk samples was conducted. The analysis between groups A and B aimed to investigate the impact of antibiotic treatment during the dry period on the mammary gland microbiota. The results indicated that, compared to group A, the abundance of *Pseudomonas* (*p* = 0.01), *unclassified Enterobacteriaceae* (*p* = 0.01), and *Serratia* (*p* = 0.01) significantly increased in the milk samples of group B. Conversely, the abundance of *Psychrobacter* (*p* = 0.04), *Romboutsia* (*p* = 0.01), *Clostridium sensu stricto 1* (*p* = 0.007), *Turicibacter* (*p* = 0.01), *Corynebacterium* (p = 0.01), *Staphylococcus* (*p* = 0.02), and others decreased (Figure 5A).

The differential analysis between groups B and C aimed to explore the changes in the mammary gland microbiota during the period from calving to the recovery of lactation in dairy cows. The species Venn diagram indicated that 50 bacterial genera were enriched in the milk samples of group B, while 604 bacterial genera were identified in the milk samples of group C, highlighting a significant increase in the diversity of the mammary gland microbiota during this period, as shown in Figure 1B. Compared to group B, the abundances of *Pseudomonas* (*p* = 0.02), *unclassified Enterobacteriaceae* (*p* = 0.01), and *Serratia* (*p* = 0.01) in group C milk samples were significantly reduced. Conversely, the abundances of Acinetobacter (*p* = 0.02), *Psychrobacter* (*p* = 0.01), *Brevundimonas* (*p* = 0.01), *Romboutsia* (*p* = 0.01), and *Turicibacter* (*p* = 0.01) increased. Although the abundance of *Staphylococcus* also increased, this change was not statistically significant (*p* = 0.06) (Figure 5B).

A differential analysis of the microbial composition between groups A and C was conducted to assess the recovery degree of the mammary gland microbiota 14 days after calving in dairy cows. Compared to the 604 bacterial genera enriched in group C milk samples, group A milk samples contained 698 bacterial genera, and the Shannon index showed no significant difference in bacterial diversity between the two groups, indicating that the diversity of the mammary gland microbiota had recovered around 14 days post-calving, as presented in Table 2 and Figure 1B. However, there were still some differences in bacterial genera between the two groups. Compared to group A, the abundance of *Acinetobacter* (*p* = 0.04), *Pseudomonas* (*p* = 0.01), and *Brevundimonas* (*p* = 0.01) in group C milk samples significantly increased, the abundance of other genera such as *Romboutsia* (*p* = 0.01), *Clostridium sensu stricto 1* (*p* = 0.01), *Turicibacter* (*p* = 0.01), *Streptococcus* (*p* = 0.02) decreased, and the abundance of *Staphylococcus* also decreased, but without significant difference (*p* = 0.14) (Figure 5C).

## 4 Discussion

The mammary environment in healthy cows, including the milk, is not sterile; rather, it features a rich and diverse microbial community, with environmental microorganisms invading through the nipple duct as the primary source of colonization (10). The use of antibiotics during the dry period in dairy cows is significant for treating potential intramammary infections and preventing new infections caused by environmental pathogens. This helps to reduce the incidence of mastitis during the dry period and the subsequent lactation. This is particularly common in high-yielding dairy cow breeds with relatively lower disease resistance, such as Holstein cows (11–13). This small-scale study conducted a preliminary assessment of the effects of dry cow therapy on the mammary microbiota and its post-calving recovery. High-throughput sequencing technology was employed to analyze milk samples collected from dairy cows at three time points before and after the dry period. Selecting the first quarter of the milk area on the left side of the cow for sampling analysis can reduce the complexity of sample processing, ensure consistency in sampling conditions, and help reduce individual differences. The results of this study only reflect the overall dynamic trend of breast microbiota before and after administration during the dry milk period, and cannot represent the variability of microbiota caused by the mutual influence between all milk regions of cows, which is also one of the limitations of the results of this study. Sequencing analysis of the V3–V4 region of the 16S rRNA gene was performed to assess the diversity of the microbial community structure in the milk samples. The *α*-diversity results indicated that significant changes in the richness and diversity of the microbial community occurred in the cow’s mammary microenvironment throughout the dry period after antibiotic treatment compared to before treatment. This change is reflected in the diminished diversity and richness of the original microbial community within the mammary gland, a significant decline that may stem from the weakened colonization capacity of mammary microbes. This phenomenon increases the risk of intramammary infection in dairy cows and further explains why dry cows exhibit heightened susceptibility to environmental pathogens during early lactation. Moreover, notably, the richness and diversity of the microbial community within the mammary gland essentially recovered by day 14 post-calving. This indicates that the mammary gland possesses a certain inherent capacity for recovery following antibiotic intervention. The relatively rapid reestablishment of the intramammary microbiota implies that a single dose of benzathine cefapirin, when employed in dry-cow therapy, does not result in a long-term dysbiosis of the mammary microbial community. This finding highlights the safety of this dry-off strategy. These findings provide insights into the rate and extent of changes in the mammary microbiota from the time of alteration during the dry period to its baseline recovery, which may be significant for developing effective management strategies.

In the study, the primary bacterial phyla in Group A, ranked by abundance, were *Firmicutes* (60%), *Proteobacteria* (21%), *Actinobacteria* (13%), and *Bacteroidetes* (6%). These dominant bacterial phyla exhibited high abundance characteristics irrespective of location, time, or stage of lactation in cows, as reflected in numerous studies (14–17). Based on the results of this study, *Firmicutes* and *Proteobacteria* were consistently the most abundant bacterial phyla across all three sampling time points, aligning with the findings of Bonsaglia ECR (18). Certain species within the *Firmicutes* phylum are involved in various nutrient metabolism and immune regulation processes, playing a crucial role in the bacterial community of healthy cow mammary glands (14, 19). The relative abundances of *Firmicutes* (5%), *Actinobacteria* (<1%), and *Bacteroidetes* (<1%) in Group B milk samples all decreased compared to Group A, with *Firmicutes* experiencing the most significant decline, dropping from 60 to 5%. This reduction suggests that antibiotics may have effectively inhibited *Firmicutes*, which include pathogens such as *Staphylococcus* and *Streptococcus* associated with mastitis. During the recovery period of the mammary gland microbiota, the relative abundance of *Firmicutes* (26%) increased but did not reach the 60% level observed in Group A. Meanwhile, *Proteobacteria* (52%) remained the dominant phylum at this stage, largely aligning with the findings of Salman MM (20) regarding the distribution of bacterial phyla in the milk of healthy cows. Interestingly, in Group B, the relative abundance of *Proteobacteria* was 95%, significantly higher than the abundance reported in the study by Patangia DV (21). Compared to the use of broad-spectrum antibiotics, narrow-spectrum antibiotics may focus more on treating and eliminating Gram-positive bacteria during the dry period. However, in studies by Derakhshani H (22) and Vasquez A (23), where narrow-spectrum antibiotics were used to treat mastitis in dairy cows, the abundance of *Proteobacteria* in colostrum was notably lower than in our findings. In Vasquez A’s study, the narrow-spectrum antibiotic used was cefapirin benzathine (ToMorrow; Boehringer Ingelheim). Therefore, the observed differences may be attributed to variations in farm location, management practices, or laboratory methodologies. In addition, the decreased abundance of *Firmicutes* and other phyla may alter the competitive dynamics with Proteus; some members of the Proteobacteria exhibit antibiotic resistance; and the characteristics of the microbial environment on farms, along with other factors, may all contribute to the relatively high abundance of Proteus (24–26), warranting further investigation.

*Psychrobacter* (9.6%) was the most abundant genus found in Group A. This genus has been reported on multiple occasions in the milk of healthy cows (27, 28). *Psychrobacter* has a wide range of environmental and biological sources; however, infections caused by this genus are rare, although some *Psychrobacter* species have been reported to cause diseases in mammals (29, 30). Catozzi C found that the relative abundance of *Psychrobacter* was associated with the health status of the mammary gland when studying the microbiota in buffalo milk under conditions of clinical mastitis (CM), subclinical mastitis (SCM), and health (31). However, there is currently no research confirming an association between *Psychrobacter* and the occurrence of bovine mastitis (27). In the study, a lower abundance of *Psychrobacter* (less than 1%) was observed only in Group B, potentially due to collateral effects of antibiotics; however, its abundance subsequently recovered to 4.3% during microbial re-establishment.

In the study, the genera *Romboutsia* (8.9%), *Clostridium sensu stricto 1* (5.7%), *Turicibacter* (5.6%), and *Corynebacterium* (5.3%) were identified as the four most abundant genera in milk samples from Group A, after *Psychrobacter*. This finding differs somewhat from the categories of dominant genera described in many studies of healthy milk samples (17, 27, 32). In Group B, the relative abundances of these genera remained below 1%, and they still did not appear as dominant genera even on day 14. Additionally, *Romboutsia* is dominant in the small intestine of ruminants, with numerous species demonstrating metabolic capacities for carbohydrate utilization, amino acid fermentation, and anaerobic respiration (33, 34). Numerous studies suggest that the genus *Romboutsia* may play a vital role in maintaining host health (35, 36) *Clostridium sensu stricto 1*, a taxonomic unit within the *Clostridium genus*, was officially proposed in 2016 to designate the group of butyrate-producing Clostridium species as *Clostridium sensu stricto 1* (37). This genus includes both beneficial and harmful species; for example, pathogenic bacteria like *Clostridium perfringens* have been found in dairy cow milk, intestines, hides, feed, drinking water, and milking parlor air. *Clostridium perfringens* in raw milk is reported to originate from environmental (38, 39). In the study, the clinical symptoms and somatic cell counts of the dairy cows indicated they were in a relatively healthy state, making it difficult to determine the specific implications of changes in *Clostridium sensu stricto* 1 abundance in milk. Therefore, we speculate that this may reflect some of the potential beneficial effects associated with this genus. *Turicibacter* is an important component of mammalian intestinal microbiota, and it has been frequently reported in the intestines of pigs, rats, and insects, as well as in whole milk. This genus is also found in cow dung and the rumen (40–42). *Turicibacter* may serve as resident microbiota in the mammary glands of dairy cows, though its precise mechanisms of action remain unclear (43). *Corynebacterium* is regarded as a secondary pathogen capable of causing mastitis, with *Corynebacterium bovis* frequently isolated from cases of subclinical mastitis (44, 45). However, *Corynebacterium* is also commonly found in milk from cows without mastitis symptoms (46), which aligns with our findings. Liu et al. (47) observed that the relative abundances of *Romboutsia*, *Clostridium sensu stricto 1, Turicibacter,* and *Corynebacterium* in the mammary microbiota decreased with increasing somatic cell counts, suggesting that these four genera may play beneficial roles in dairy cow mammary glands. Based on the somatic cell results in this study, we infer that these genera likely play similar roles. In our study, the abundances of these genera were significantly reduced following antibiotic administration, indicating that the treatment diminished both potential pathogens and some beneficial commensals, which may lead to short-term instability of the mammary microbiota during the dry period. However, by 14 days post-calving, these genera showed partial recovery, though not to pre-dry-off levels.

The study also identified the genus *Acinetobacter*, which was the most abundant genus in the samples from Group C. In the study by Patangia DV (21), *Acinetobacter* was consistently the most abundant genus at multiple time points after lactation resumed in cows treated with antibiotics during the dry period, which aligns with the observations in this study. *Acinetobacter* is a common environmental microorganism capable of invading mammary glands through damaged teats or milk ducts (48). Although the pathogenic mechanisms of certain environmentally derived microorganisms in the mammary gland are not fully understood, these organisms often produce virulence factors that can induce opportunistic infections in immunocompromised hosts. Additionally, studies have indicated that milk from healthy cows contains more *Acinetobacter* than milk from cows with mastitis. A negative correlation was also observed between the abundances of *Lactococcus* and *Acinetobacter*, suggesting a potential competitive inhibition relationship between these two genera (49). This finding is consistent with the results of this study, as we observed fewer *Lactococcus* and more *Acinetobacter* in Group C compared to Group A. Moreover, certain *Lactococcus* variants have been reported to cause mastitis in cattle (50). Therefore, the observation of such results under healthy conditions may reflect the beneficial role of *Acinetobacter* in the mammary gland.

*Staphylococcus* and *Streptococcus* are the primary pathogenic groups responsible for clinical and subclinical mastitis in dairy cows (51). In the study, the abundances of *Staphylococcus* and *Streptococcus* in the milk samples from Group B were significantly lower than those in Group A. The abundances of these two genera in Group C were also lower than those in Group A. In summary, the use of antibiotics before drying off reduced the abundance of *Staphylococcus* and *Streptococcus* in the milk samples after calving, despite the results for *Staphylococcus* showing no significant difference. Mammary infections during the dry period are a primary cause of clinical mastitis in most dairy cows. We found that this dry-cow therapy helps to sustain the suppression of mastitis pathogens such as *Staphylococcus aureus* and *Streptococcus* spp. after calving. This indicates that the application of this dry-off protocol may effectively block the transmission of pathogens into early lactation, thereby contributing to mastitis prevention. This has positive implications for preventing mastitis during the dry period and after calving.

Additionally, it was observed that the abundance of *Lactobacillus* in milk samples from Group C (0.03%) was higher than in Group A (0.007%). Conversely, the abundance of *Bifidobacterium* was lower in Group C than in Group A. These genera typically serve as beneficial bacteria in raw milk, yogurt, and cheese (52, 53).

## 5 Conclusion

Understanding the mammary microbiota before and after treatment during the dry period in dairy cows is essential for maintaining their health and productivity. High-throughput sequencing of the V3–V4 variable regions of the 16S rRNA gene enables analysis of the mammary microbial communities, facilitating the development of preventive and therapeutic strategies for the dry period in dairy cows. The study demonstrates that a single intramammary infusion of cephapirin benzathine, administered during the dry period, reduces the abundance of various taxonomic groups in the first milk after calving. These include *Firmicutes*, *Actinobacteria*, *Bacteroidetes*, and genera such as *Psychrobacter*, *Romboutsia*, *Clostridium sensu stricto 1*, *Turicibacter*, *Corynebacterium*, *Staphylococcus*, *Streptococcus*, and *Pseudonocardia*. The findings also indicate that the diversity and richness of the mammary microbiota are largely restored approximately 14 days after calving. Additionally, antibiotic treatment increased the abundance of beneficial bacteria, such as *Lactobacillus*. In summary, this exploratory study preliminarily indicates the effects of Cefapirin Benzathine Intramammary Infusion (Dry Period) infusion during the dry period on the mammary microbiota in dairy cows and predicts the approximate time required for the mammary microbiota to return to pre-dry period levels following antibiotic treatment during the dry period.

## Data Availability

The raw data supporting the conclusions of this article will be made available by the authors, without undue reservation.

## Footnotes

1
https://www.ema.europa.eu/en/veterinary-regulatory-overview/antimicrobial-resistance-veterinary-medicine/european-surveillance-veterinary-antimicrobial-consumption-esvac-2009-2023

2
https://vetbooks.ir/laboratory-handbook-on-bovine-mastitis-3rd-edition/
